# Telemedicine in the COVID-19 Era: A chance to make a better tomorrow

**DOI:** 10.12669/pjms.36.6.3112

**Published:** 2020

**Authors:** Uzma Zubair Khan

**Affiliations:** 1 Prof. Uzma Zubair Khan. M.D Department of Medicine University of Missouri-Columbia, Cosmopolitan International Diabetes and Endocrinology Center, One Hospital Drive, Columbia, Missouri, 65212, USA

**Keywords:** Telemedicine, COVID-19, Pakistan, Challenges

## Abstract

Telemedicine use is increasing globally and in Pakistan. However, Pakistan faces unique challenges related to socioeconomic, geographic and perhaps political challenges. This is the time for Pakistan to create policies and protocols for ethical and efficient use of telemedicine. The goal of this manuscript is to start a discussion, by encouraging questions, and identifying challenges for healthcare providers.

## INTRODUCTION

Pakistan is a densely populated developing country, facing multiple challenges in the healthcare field. A major challenge is access to healthcare especially due to geographic, cultural and socioeconomic barriers.^1^ The COVID-19 pandemic is more than a health emergency; it is an economic and social emergency and has brought health disparities to the forefront. There are many questions being asked about the expanding role of technology in this time of social distancing. Raising the right questions related to use of technology in healthcare and making sure we are aware of the challenges ahead may create an opportunity for success in Pakistan. The recent surge in telemedicine in Pakistan occurred because of fear of contracting COVID-19 and stress on social distancing. Telemedicine had already been gaining popularity in Pakistan for many years, with a significant contribution from both nonprofits^2^, and academic centers.^3^ In the last few months, social distancing has led to this technology becoming even more important as a support system and in the increasing use of telemedicine.

### Ask the right questions

### Define the problem

When we talk about telemedicine as the solution to the problem, do we really understand what the problem is? Studies assessing access to health in Pakistan are limited, and mainly focus on economic barriers.^4^ The short-term surge in telemedicine use during the COVID-19 pandemic may not persist to become a long-term solution unless an effort is made to identify expected outcomes that will lead to a decrease in healthcare delivery disparity rather than widen the gap for provision of healthcare services for people with limited access.

### The doctor-patient relationship

Another important question should be, what about the doctor-patient relationship? We have all heard about the toddler who thought his grandfather was an iPad. It is important to visualize a “virtual “relationship between doctors and patients based on cultural sensitivity in Pakistan today. Addressing the expectations of both doctors and patients is needed for acceptance of such a relationship in the future.

**Table-I:** Questions to ask regarding telemedicine.

The problem:	What is the problem?
	Is this a short term fix or investment in a long term solution?
	What is the expected outcome?
The team:	Are we ready to redefine the doctor-patient relationship?
	What are the patient expectations?
The visit:	Do all participants understand a virtual visit?
	Is the appropriate technology in place?

### More than a video visit

All participants need to understand the various forms of technology-based health communications.^5^ When we talk about telemedicine as a component of patient care, it is important to keep in mind not just access to the technology in the community, but also issues related to privacy, communication and distant learning as important for successful programs.

### Identify the challenges

There is no simple way to address the challenges presented by telemedicine. Looking at these challenges as related to patients, providers, and the healthcare system may be a good starting point.

### Challenges for patients

Patient challenges include access to technology, acceptance of technology, relationship with the provider and level of health literacy. Healthcare is complicated and diagnoses can be difficult for patients to understand. For example, diabetes mellitus is a complex disease and its long-term management is more than blood glucose control. A clinic visit for chronic diabetes management is challenging in the best of circumstances, but becomes an even bigger challenge during a telemedicine visit.^6^ During an in-person visit, the patient can talk, discuss blood glucose and be examined at the same time. Education about medication side effects and compliance is done at the same time as dose adjustment and prescription writing. During a telemedicine visit this efficiency may be lost. Lack of a detailed physical exam may miss important findings like decreased of sensations or a new heart murmur. Ensuring patient privacy and addressing patient concerns to maintain a high level of patient satisfaction will continue to be challenging.

### Challenges for doctors

Doctors face many challenges in Pakistan, but when it comes to telemedicine it is naïve to assume that a doctor who has years of clinical practice will be able to start telemedicine visits in a day. Provider challenges include practicing “No-Touch” medicine, time management, building team relationships and technology literacy for doctors, especially those trained many years ago. It is important to identify the challenge for providers to maintain professional well-being while maintaining medical knowledge and keeping pace with the progressively increasing medical information. The instincts that are so useful in the clinic to identify a patient who looks sick with cold and clammy skin may be lost in a virtual visit creating a challenge for even experienced clinicians to learn a new way to practice medicine.

A telemedicine visit may not be less time consuming for doctors and time management will be crucial in a telemedicine visit with fixed start and stop times. The format for the visit may change as we learn more, but changing from open ended, conversational questions to focused questions could lead to a fragmented, impersonal interaction. The workflow for a telemedicine visit will need to be redefined including pre-visit appointment set up, organization of records for review, streamlining the interactive visit, and development of a post visit protocol to ensure follow-up on testing and patient calls. Data storage, access and privacy are challenges that will also need to be tackled to ensure success.

**Table-II:** Challenges for telemedicine.

Challenges for patients	Access to technology
	Acceptance of technology
	Relationship with provider
	Health literacy of the patient
Challenges for doctors	Practicing “No-Touch” medicine
	Time management
	Building team relationships
	Technology literacy of the doctor
	Maintenance of professional wellbeing
	Maintenance of medical knowledge
Challenges for healthcare system	Policy development, adherence and monitoring
	Define liability
	Accreditation and licensure of physicians
	Establish reimbursement systems
	Create stable information technology network, hardware and personnel support
	Ensure long term investment

Technology literacy, or the ability to work with up to date technology independently as well as part of a team simultaneously, is a big challenge for providers, many of whom were not trained in electronic medical records until the last decade. This is important to realize, especially in chronic conditions where formulating a care plan is complex, and patient education and adherence is a requirement. Continued medical education and licensure, which have traditionally been based on clinical knowledge and skills, will now need to include technology skills. This is an area new to the practice of medicine, and will take years to mature. Most importantly, learning these new skills while maintaining and keeping pace with progressively increasing medical information may create another stressor for doctors.

### Challenges for the healthcare system

For Pakistan, this is the time to address use of technology in healthcare and involve clinicians, public health and information technology experts to identify the many challenges ahead.^7^ The major challenge will be policy development, and establishing a system for adherence and monitoring. Accreditation and licensure of physicians, setting up reliable reimbursement systems and defining liability may need major changes to the existing system. In addition information technology network, hardware and trained personnel will be needed to ensure long term success in telemedicine.

In general, Pakistan lacks a focus on health policy^8^ at every level. Telemedicine, with the use of information technology may become the stimulus needed for a review of health policy at government and hospital level. It is important to define who will make the policies, determine investment in hardware and personal, and evaluate the impact of programs. Involving the healthcare community will be instrumental in ensuring adherence, monitoring, and liability determined by these policies.

There are many privacy concerns in the field of healthcare, but in telemedicine has unique challenges regarding cyber security related to internet connection, chosen platform and visit location. There are also many liability issues, especially given the complexity of data involved, socioeconomic and cultural issues that need to be defined.

### Time to Change

By asking the right questions and identifying the challenges, telemedicine may be the much needed tool for improving healthcare in Pakistan. Over the last few years, it has been encouraging to see more doctors using telemedicine and the involvement of nonprofits and academic centers. Telemedicine is a team effort, and involves building trust and confidence. The focus on strengthening doctor-patient relationship and building a healthcare network to improve access as a common goal will benefit all partners.

The increasing prevalence of diseases like uncontrolled diabetes mellitus with significant comorbidities like obesity and hypertension can benefit from telemedicine. The COVID-19 pandemic has created an unexpected opportunity for Pakistan to address many of the challenges in healthcare via telemedicine. This unique experience can create programs that are designed for implementation in Pakistan with distinctive cultural, socioeconomic and geographical needs. Faced with a common threat that is blind to wealth, gender and social status, we can create the equity in healthcare we have craved. This is the time to educate patients, doctors and the community. This “Once-in- a- Century Pandemic”^9^ is our chance to change the healthcare delivery model in Pakistan. This is the time when we, as healthcare professionals can face our vulnerabilities head-on and choose to be compassionate catalysts for a better tomorrow.
